# 5-(3,4,5-Trimeth­oxy­phen­yl)-1,3,4-oxadiazole-2(3*H*)-thione

**DOI:** 10.1107/S1600536810024967

**Published:** 2010-07-03

**Authors:** Aamer Saeed, Muhammad Akram, Abdul Rauf, Michael Bolte

**Affiliations:** a Department of Chemistry, Quaid-i-Azam University, Islamabad 45320, Pakistan; b Department of Chemistry, Islamia University Bhawalpur, Pakistan; c Institut für Anorganische Chemie, J. W. Goethe-Universität Frankfurt, Max-von-Laue-Strasse 7, 60438 Frankfurt/Main, Germany

## Abstract

The two rings in the title compound, C_11_H_12_N_2_O_4_S, are roughly coplanar [dihedral angle = 6.77 (8)°]. Whereas the two outer methyl groups of the three meth­oxy groups are almost coplanar with the aromatic ring to which they are attached [C—C—O—C torsion angles = 8.5 (3) and −8.3 (3)°], the methyl group of the central meth­oxy substituent is not [C—C—C—C = −78.4 (3)°]. The crystal packing is stabilized by N—H⋯O hydrogen bonding.

## Related literature

For background to the use of 1,3,4-oxadiazo­les, see: Erden *et al.* (2005 ▶); Smicius *et al.* (2002 ▶); Dutta & Kataky (1992 ▶). For details of the biological activity of 1,3,4-oxadiazo­les, see: Chen, *et al.* (2000 ▶); Mehuskiene, *et al.* (2003 ▶); El-Emam *et al.* (2004 ▶); Krasovshii *et al.* (2000 ▶).
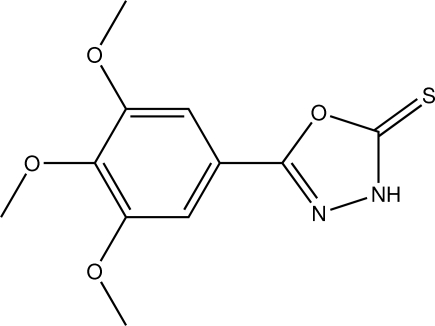


## Experimental

### 

#### Crystal data

C_11_H_12_N_2_O_4_S
*M* *_r_* = 268.29
Monoclinic, 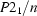

*a* = 12.506 (2) Å
*b* = 7.1577 (7) Å
*c* = 13.451 (2) Å
β = 96.558 (12)°
*V* = 1196.2 (3) Å^3^
*Z* = 4
Mo *K*α radiation
μ = 0.28 mm^−1^
*T* = 173 K
0.37 × 0.33 × 0.32 mm

#### Data collection

Stoe IPDS II two-circle diffractometer
Absorption correction: multi-scan (*MULABS*; Spek, 2009 ▶; Blessing, 1995 ▶) *T*_min_ = 0.904, *T*_max_ = 0.916
7121 measured reflections
2235 independent reflections
1679 reflections with *I* > 2σ(*I*)
*R*_int_ = 0.075

#### Refinement

*R*[*F* ^2^ > 2σ(*F* ^2^)] = 0.044
*wR*(*F* ^2^) = 0.112
*S* = 0.94
2235 reflections
171 parameters
H atoms treated by a mixture of independent and constrained refinement
Δρ_max_ = 0.45 e Å^−3^
Δρ_min_ = −0.27 e Å^−3^

### 

Data collection: *X-AREA* (Stoe & Cie, 2001 ▶); cell refinement: *X-AREA*; data reduction: *X-AREA*; program(s) used to solve structure: *SHELXS97* (Sheldrick, 2008 ▶); program(s) used to refine structure: *SHELXL97* (Sheldrick, 2008 ▶); molecular graphics: *XP* (Sheldrick, 2008 ▶); software used to prepare material for publication: *SHELXL97*.

## Supplementary Material

Crystal structure: contains datablocks global, I. DOI: 10.1107/S1600536810024967/tk2682sup1.cif

Structure factors: contains datablocks I. DOI: 10.1107/S1600536810024967/tk2682Isup2.hkl

Additional supplementary materials:  crystallographic information; 3D view; checkCIF report

## Figures and Tables

**Table 1:** Hydrogen-bond geometry (Å, °)

*D*—H⋯*A*	*D*—H	H⋯*A*	*D*⋯*A*	*D*—H⋯*A*
N1—H1⋯O17^i^	0.90 (3)	2.06 (3)	2.881 (2)	151 (2)

Symmetry code: (i) 
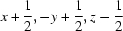
.

**Fig. 1.**
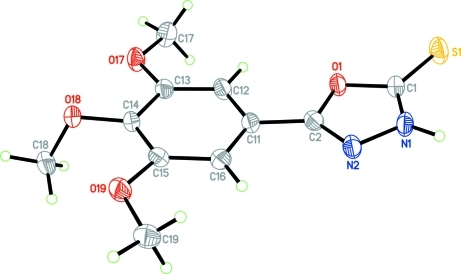
Molecular structure of title compound. Displacement ellipsoids are drawn at the 50% probability level.

## supplementary crystallographic information

### Comment

Substituted 1,3,4-oxadiazoles attract interest in materials science due to their important applications in industrial, agricultural and polymer chemistry (Erden *et al.*, 2005, Smicius *et al.*, 2002, Dutta & Kataky, 1992) and their wide range of biological activities, such as bactericidal, anti-inflammatory, antiviral, antimicrobial, tuberculostatic, anti convulsive and fungicidal activities (Chen *et al.*, 2000; Mehuskiene *et al.*, 2003; El-Emam *et al.*, 2004; Krasovshii *et al.*, 2000). The title compound was prepared by refluxing 3,4,5-trimethoxybenzohydrazide and potassium hydroxide with carbon disulfide in ethanol.

The two rings in the title compound, C_11_H_12_N_2_O_4_S, are coplanar [dihedral angle = 6.77 (8) °]. Whereas the two outer methyl groups of the three methoxy groups are coplanar with the aromatic ring to which they are attached [C—C—O—C torsion angles = 8.5 (3) and -8.3 (3) °], the methyl group of the central methoxy substituent is not [C—C—C—C = -78.4 (3) °]. The crystal packing is stabilized by N—H···O hydrogen bonding.

### Experimental

A mixture of 3,4,5-trimethoxybenzohydrazide (0.03 mol) and potassium hydroxide (0.03 mol) was dissolved in ethanol, followed by addition of carbon disulfide (0.08 mol) drop wise under stirring. The reaction mixture was heated under reflux for 14 h. After completion of reaction as indicated by TLC, the reaction mixture was concentrated and residue was dissolved in H_2_O, filtered and acidified with dilute hydrochloric acid (pH = 2–3). The resulting precipitate was filtered and recrystallized from ethanol to give crystalline solid in 82% yield; m.p. = 453–455 K. IR (KBr, cm^-1^), 3227–3012 (NH), 2939 (Ar—CH), 1581 (Cδb N), 1503, 1454, 1447 (Cδb C, aromatic), 1359 (C═S), 1233–1163 (C—O—C).

### Refinement

H atoms were located in a difference Fourier map. The H atom bonded to N was freely refined. The H atoms bonded to C were refined using a riding model with isotropic displacement parameters *U*_iso_(H) set to 1.2*U*_eq_(C) and with C—H = 0.95 Å or *U*_iso_(H) set to 1.5*U*_eq_(C_methyl_) and with C—H = 0.98 Å. The methyl groups were allowed to rotate but not to tip.

### Crystal data

**Table:**

C_11_H_12_N_2_O_4_S	*F*(000) = 560
*M**_r_* = 268.29	*D*_x_ = 1.490 Mg m^−^^3^
Monoclinic, *P*2_1_/*n*	Mo *K*α radiation, λ = 0.71073 Å
Hall symbol: -P 2yn	Cell parameters from 5950 reflections
*a* = 12.506 (2) Å	θ = 3.6–25.9°
*b* = 7.1577 (7) Å	µ = 0.28 mm^−^^1^
*c* = 13.451 (2) Å	*T* = 173 K
β = 96.558 (12)°	Block, colourless
*V* = 1196.2 (3) Å^3^	0.37 × 0.33 × 0.32 mm
*Z* = 4	

### Data collection

**Table:**

Stoe IPDS II two-circle diffractometer	2235 independent reflections
Radiation source: fine-focus sealed tube	1679 reflections with *I* > 2σ(*I*)
graphite	*R*_int_ = 0.075
ω scans	θ_max_ = 25.7°, θ_min_ = 3.5°
Absorption correction: multi-scan (*MULABS*; Spek, 2009; Blessing, 1995)	*h* = −12→15
*T*_min_ = 0.904, *T*_max_ = 0.916	*k* = −7→8
7121 measured reflections	*l* = −16→16

### Refinement

**Table:**

Refinement on *F*^2^	Secondary atom site location: difference Fourier map
Least-squares matrix: full	Hydrogen site location: inferred from neighbouring sites
*R*[*F*^2^ > 2σ(*F*^2^)] = 0.044	H atoms treated by a mixture of independent and constrained refinement
*wR*(*F*^2^) = 0.112	*w* = 1/[σ^2^(*F*_o_^2^) + (0.0699*P*)^2^] where *P* = (*F*_o_^2^ + 2*F*_c_^2^)/3
*S* = 0.94	(Δ/σ)_max_ < 0.001
2235 reflections	Δρ_max_ = 0.45 e Å^−^^3^
171 parameters	Δρ_min_ = −0.27 e Å^−^^3^
0 restraints	Extinction correction: *SHELXL97* (Sheldrick, 2008), Fc^*^=kFc[1+0.001xFc^2^λ^3^/sin(2θ)]^-1/4^
Primary atom site location: structure-invariant direct methods	Extinction coefficient: 0.024 (3)

### Special details

**Table:**

Geometry. All e.s.d.'s (except the e.s.d. in the dihedral angle between two l.s. planes) are estimated using the full covariance matrix. The cell e.s.d.'s are taken into account individually in the estimation of e.s.d.'s in distances, angles and torsion angles; correlations between e.s.d.'s in cell parameters are only used when they are defined by crystal symmetry. An approximate (isotropic) treatment of cell e.s.d.'s is used for estimating e.s.d.'s involving l.s. planes.
Refinement. Refinement of *F*^2^ against ALL reflections. The weighted *R*-factor *wR* and goodness of fit *S* are based on *F*^2^, conventional *R*-factors *R* are based on *F*, with *F* set to zero for negative *F*^2^. The threshold expression of *F*^2^ > σ(*F*^2^) is used only for calculating *R*-factors(gt) *etc*. and is not relevant to the choice of reflections for refinement. *R*-factors based on *F*^2^ are statistically about twice as large as those based on *F*, and *R*- factors based on ALL data will be even larger.

### Fractional atomic coordinates and isotropic or equivalent isotropic displacement parameters (Å^2^)

**Table:**

	*x*	*y*	*z*	*U*_iso_*/*U*_eq_	
S1	0.40007 (5)	0.10396 (10)	0.11859 (4)	0.0358 (2)	
O1	0.40507 (12)	0.1537 (2)	0.31509 (9)	0.0258 (4)	
C1	0.45909 (18)	0.1504 (3)	0.23147 (14)	0.0260 (5)	
N1	0.56078 (16)	0.1912 (3)	0.26552 (13)	0.0278 (4)	
H1	0.619 (2)	0.211 (4)	0.233 (2)	0.040 (7)*	
N2	0.57512 (15)	0.2258 (3)	0.36818 (12)	0.0273 (4)	
C2	0.48040 (17)	0.2018 (3)	0.39406 (14)	0.0234 (5)	
C11	0.44138 (17)	0.2288 (3)	0.49170 (14)	0.0232 (5)	
C12	0.33315 (17)	0.2074 (3)	0.49941 (14)	0.0248 (5)	
H12	0.2854	0.1702	0.4428	0.030*	
C13	0.29410 (17)	0.2409 (3)	0.59106 (14)	0.0234 (5)	
C14	0.36466 (18)	0.3006 (3)	0.67290 (14)	0.0238 (5)	
C15	0.47369 (17)	0.3228 (3)	0.66338 (14)	0.0239 (5)	
C16	0.51427 (18)	0.2840 (3)	0.57291 (14)	0.0246 (5)	
H16	0.5890	0.2948	0.5669	0.030*	
O17	0.18925 (12)	0.2223 (2)	0.60687 (10)	0.0289 (4)	
O18	0.32183 (13)	0.3468 (2)	0.75958 (10)	0.0288 (4)	
O19	0.53524 (12)	0.3899 (2)	0.74645 (10)	0.0307 (4)	
C17	0.11725 (18)	0.1399 (4)	0.52800 (16)	0.0336 (6)	
H17A	0.1079	0.2252	0.4707	0.050*	
H17B	0.0473	0.1169	0.5520	0.050*	
H17C	0.1474	0.0215	0.5075	0.050*	
C18	0.34058 (19)	0.2114 (4)	0.83861 (15)	0.0328 (6)	
H18A	0.3103	0.0907	0.8153	0.049*	
H18B	0.3061	0.2531	0.8966	0.049*	
H18C	0.4182	0.1980	0.8578	0.049*	
C19	0.64872 (18)	0.3968 (4)	0.74341 (16)	0.0335 (6)	
H19A	0.6768	0.2696	0.7393	0.050*	
H19B	0.6830	0.4575	0.8042	0.050*	
H19C	0.6644	0.4682	0.6846	0.050*	

### Atomic displacement parameters (Å^2^)

**Table:**

	*U*^11^	*U*^22^	*U*^33^	*U*^12^	*U*^13^	*U*^23^
S1	0.0358 (4)	0.0522 (4)	0.0195 (3)	−0.0034 (3)	0.0042 (2)	−0.0050 (2)
O1	0.0240 (8)	0.0374 (10)	0.0168 (7)	−0.0006 (7)	0.0050 (5)	−0.0010 (6)
C1	0.0267 (12)	0.0313 (13)	0.0212 (10)	0.0023 (10)	0.0076 (8)	0.0022 (8)
N1	0.0250 (10)	0.0401 (12)	0.0196 (8)	0.0001 (9)	0.0086 (7)	−0.0011 (8)
N2	0.0262 (10)	0.0376 (12)	0.0186 (8)	0.0003 (8)	0.0050 (7)	0.0000 (7)
C2	0.0231 (11)	0.0263 (12)	0.0208 (9)	0.0004 (9)	0.0018 (8)	0.0015 (8)
C11	0.0273 (11)	0.0243 (12)	0.0186 (9)	0.0029 (9)	0.0051 (8)	0.0017 (8)
C12	0.0249 (11)	0.0317 (13)	0.0179 (9)	−0.0018 (10)	0.0021 (8)	−0.0004 (8)
C13	0.0218 (11)	0.0284 (12)	0.0203 (10)	0.0009 (9)	0.0037 (8)	0.0013 (8)
C14	0.0281 (12)	0.0278 (12)	0.0161 (9)	0.0046 (9)	0.0046 (8)	−0.0004 (8)
C15	0.0252 (11)	0.0265 (11)	0.0190 (9)	0.0027 (9)	−0.0012 (8)	0.0010 (8)
C16	0.0213 (11)	0.0289 (13)	0.0239 (10)	0.0018 (9)	0.0038 (8)	0.0021 (8)
O17	0.0209 (8)	0.0453 (10)	0.0212 (7)	−0.0042 (7)	0.0053 (6)	−0.0041 (6)
O18	0.0321 (9)	0.0370 (10)	0.0183 (7)	0.0087 (7)	0.0064 (6)	−0.0004 (6)
O19	0.0245 (8)	0.0438 (10)	0.0228 (7)	0.0001 (7)	−0.0020 (6)	−0.0049 (6)
C17	0.0250 (12)	0.0430 (16)	0.0332 (12)	−0.0056 (11)	0.0043 (9)	−0.0109 (10)
C18	0.0358 (13)	0.0436 (15)	0.0197 (10)	0.0028 (11)	0.0060 (9)	0.0018 (9)
C19	0.0249 (12)	0.0435 (15)	0.0306 (11)	−0.0001 (11)	−0.0034 (9)	−0.0019 (10)

### Geometric parameters (Å, °)

**Table:**

S1—C1	1.645 (2)	C15—O19	1.369 (2)
O1—C1	1.377 (2)	C15—C16	1.399 (3)
O1—C2	1.381 (2)	C16—H16	0.9500
C1—N1	1.334 (3)	O17—C17	1.437 (3)
N1—N2	1.394 (2)	O18—C18	1.438 (3)
N1—H1	0.90 (3)	O19—C19	1.425 (3)
N2—C2	1.284 (3)	C17—H17A	0.9800
C2—C11	1.465 (3)	C17—H17B	0.9800
C11—C12	1.378 (3)	C17—H17C	0.9800
C11—C16	1.398 (3)	C18—H18A	0.9800
C12—C13	1.398 (3)	C18—H18B	0.9800
C12—H12	0.9500	C18—H18C	0.9800
C13—O17	1.359 (3)	C19—H19A	0.9800
C13—C14	1.397 (3)	C19—H19B	0.9800
C14—O18	1.378 (2)	C19—H19C	0.9800
C14—C15	1.393 (3)		
			
C1—O1—C2	106.11 (16)	C14—C15—C16	120.90 (18)
N1—C1—O1	104.66 (17)	C11—C16—C15	117.8 (2)
N1—C1—S1	132.15 (17)	C11—C16—H16	121.1
O1—C1—S1	123.19 (17)	C15—C16—H16	121.1
C1—N1—N2	112.87 (18)	C13—O17—C17	117.32 (16)
C1—N1—H1	131.5 (16)	C14—O18—C18	114.68 (17)
N2—N1—H1	115.3 (16)	C15—O19—C19	117.23 (17)
C2—N2—N1	103.10 (17)	O17—C17—H17A	109.5
N2—C2—O1	113.23 (17)	O17—C17—H17B	109.5
N2—C2—C11	129.62 (18)	H17A—C17—H17B	109.5
O1—C2—C11	117.04 (18)	O17—C17—H17C	109.5
C12—C11—C16	122.09 (18)	H17A—C17—H17C	109.5
C12—C11—C2	118.92 (18)	H17B—C17—H17C	109.5
C16—C11—C2	118.92 (19)	O18—C18—H18A	109.5
C11—C12—C13	119.51 (18)	O18—C18—H18B	109.5
C11—C12—H12	120.2	H18A—C18—H18B	109.5
C13—C12—H12	120.2	O18—C18—H18C	109.5
O17—C13—C14	116.15 (18)	H18A—C18—H18C	109.5
O17—C13—C12	124.19 (18)	H18B—C18—H18C	109.5
C14—C13—C12	119.6 (2)	O19—C19—H19A	109.5
O18—C14—C15	121.99 (18)	O19—C19—H19B	109.5
O18—C14—C13	117.95 (19)	H19A—C19—H19B	109.5
C15—C14—C13	119.95 (18)	O19—C19—H19C	109.5
O19—C15—C14	115.45 (18)	H19A—C19—H19C	109.5
O19—C15—C16	123.6 (2)	H19B—C19—H19C	109.5
			
C2—O1—C1—N1	1.4 (2)	C12—C13—C14—O18	174.8 (2)
C2—O1—C1—S1	−178.55 (17)	O17—C13—C14—C15	179.80 (19)
O1—C1—N1—N2	−1.4 (3)	C12—C13—C14—C15	−1.3 (3)
S1—C1—N1—N2	178.49 (19)	O18—C14—C15—O19	1.2 (3)
C1—N1—N2—C2	0.9 (3)	C13—C14—C15—O19	177.2 (2)
N1—N2—C2—O1	0.1 (2)	O18—C14—C15—C16	−176.7 (2)
N1—N2—C2—C11	−175.9 (2)	C13—C14—C15—C16	−0.7 (3)
C1—O1—C2—N2	−0.9 (2)	C12—C11—C16—C15	−1.8 (3)
C1—O1—C2—C11	175.60 (19)	C2—C11—C16—C15	175.1 (2)
N2—C2—C11—C12	175.1 (2)	O19—C15—C16—C11	−175.5 (2)
O1—C2—C11—C12	−0.8 (3)	C14—C15—C16—C11	2.2 (3)
N2—C2—C11—C16	−1.9 (4)	C14—C13—O17—C17	−172.7 (2)
O1—C2—C11—C16	−177.8 (2)	C12—C13—O17—C17	8.5 (3)
C16—C11—C12—C13	−0.2 (3)	C15—C14—O18—C18	−78.4 (3)
C2—C11—C12—C13	−177.1 (2)	C13—C14—O18—C18	105.6 (2)
C11—C12—C13—O17	−179.4 (2)	C14—C15—O19—C19	173.8 (2)
C11—C12—C13—C14	1.8 (3)	C16—C15—O19—C19	−8.3 (3)
O17—C13—C14—O18	−4.1 (3)		

### Hydrogen-bond geometry (Å, °)

**Table:**

*D*—H···*A*	*D*—H	H···*A*	*D*···*A*	*D*—H···*A*
N1—H1···O17^i^	0.90 (3)	2.06 (3)	2.881 (2)	151 (2)

Symmetry codes: (i) *x*+1/2, −*y*+1/2, *z*−1/2.
